# Biological Manganese Removal by Novel Halotolerant Bacteria Isolated from River Water

**DOI:** 10.3390/biom10060941

**Published:** 2020-06-22

**Authors:** Van Khanh Nguyen, Myung-Gyu Ha, Ho Young Kang, Dinh Duc Nguyen

**Affiliations:** 1 Laboratory of Advanced Materials Chemistry, Advanced Institute of Materials Science, Ton Duc Thang University, Ho Chi Minh City, Vietnam; nguyenvankhanh@tdtu.edu.vn; 2 Faculty of Applied Sciences, Ton Duc Thang University, Ho Chi Minh City, Vietnam; 3 Korea Basic Science Institute, Busan Center, Busan 46742, Korea; mkha@kbsi.re.kr; 4 Department of Microbiology, Pusan National University, Busan 46241, Korea; hoykang@pusan.ac.kr; 5 Institution of Research and Development, Duy Tan University, Da Nang 550000, Vietnam

**Keywords:** bioprecipitation, aerobic, river, bioremediation, biogenic Mn oxides

## Abstract

Manganese-oxidizing bacteria have been widely investigated for bioremediation of Mn-contaminated water sources and for production of biogenic Mn oxides that have extensive applications in environmental remediation. In this study, a total of 5 Mn-resistant bacteria were isolated from river water and investigated for Mn removal. Among them, *Ochrobactrum* sp. NDMn-6 exhibited the highest Mn removal efficiency (99.1%). The final precipitates produced by this strain were defined as a mixture of Mn_2_O_3_, MnO_2_, and MnCO_3_. Optimal Mn-removal performance by strain NDMn-6 was obtained at a temperature range of 25–30 °C and the salinity of 0.1–0.5%. More interestingly, strain NDMn-6 could be resistant to salinities of up to 5%, revealing that this strain could be possibly applied for Mn remediation of high salinity regions or industrial saline wastewaters. This study also revealed the potential of self-detoxification mechanisms, wherein river water contaminated with Mn could be cleaned by indigenous bacteria through an appropriate biostimulation scheme.

## 1. Introduction

Manganese, which is the chemical element with the atomic number 25, is in the first transition series in the periodic T of elements. Manganese is an abundant element in the Earth’s crust (0.1%), at approximately one-fiftieth the amount of iron [1]. In nature, Mn does not exist as a free element, but occurs in more than 100 natural minerals, mainly in the form of oxides, carbonates, and silicates [1,2]. Manganese has a total of eight oxidation states, including 0, +1, +2, +3, +4, +5, +6, and +7. However, among them, only Mn in the +2 oxidation state Mn(II) can exist as a free ion in aqueous solutions and natural water systems. The average concentration of Mn(II) in natural freshwater has been determined to be 8 µg/L [3].

Manganese has various applications in industries and in biological systems. In industry, Mn is used mainly in metallurgy and chemical production. In biological systems, Mn is an important microelement for microorganisms, plants, and humans [4]. It contributes as a key activator of numerous enzymes that catalyze several reactions in living organisms [4]. However, Mn becomes a toxic agent upon overexposure of an organism at high levels [5,6]. Excess Mn uptake to plants induces deficiencies in iron, magnesium, and calcium, because Mn competes with iron and magnesium uptake and also inhibits the translocation of calcium in the shoot apex [7]. In humans, overexposure and uptake of Mn could cause some symptoms, such as headache, insomnia, exaggerated tendon reflexes, hyper-myotonia, and memory loss, with an incidence frequency of more than 75% [8]. The World Health Organization has set the acceptable level for Mn in drinking water to be below 0.05 mg/L [9].

Contamination sources of Mn could be the natural weathering of mother rock, or anthropogenic sources such as metal processing facilities and chemical industries [10]. Currently, various technologies for Mn removal from water sources, such as ion exchange, sorption, catalytic oxidation, and biological oxidation, are available for use [11]. Among them, biological methods are preferable because of their low-cost and eco-friendliness. In these biological processes, the soluble Mn(II) is biologically oxidized to Mn(III) and Mn(IV), which subsequently precipitate as biogenic Mn oxides. Up to now, several studies that proved Mn removal by bacteria as pure cultures and as mixed consortia have been conducted [12,13,14,15,16]. Some bacterial strains that could perform well in Mn oxidation at low pH and produce high Mn removal efficiency have also been isolated [16,17,18,19]. The distribution of Mn removal by these isolated Mn(II)-oxidizing bacteria has been extensively clarified by a sequential extraction procedure [16,17].

This study aimed to isolate Mn-resistant bacteria from river water. These novel bacteria were screened for Mn-oxidizing ability focusing on Mn removal performance with high initial Mn concentration. The bacterium with the best performance was investigated for optimal Mn removal at various temperatures and salinities. The microscopic structure and crystal phases of the precipitated product obtained from bacterial Mn removal were also characterized.

## 2. Materials and Methods

### 2.1. Bacterial Enrichment and Isolation

The river water used for bacterial enrichment and isolation was collected from the Nakdong River, which flows through the Busan area. Two growth media, including nutrient broth medium and basal salt medium, were included in the enrichment process. Nutrient broth medium (NB) [20] contains 10 g/L of peptone, 5 g/L of yeast, and 5 g/L of NaCl. Basal salt medium (BSM) [21] contains 0.1 g/L of NaCl, 0.1 g/L of KH_2_PO_4_, 0.2 g/L of CaCl_2_, 0.24 g/L of NH_4_Cl, 0.12 g/L of MgCl_2_.6H_2_O, 0.5 g/L of yeast extract, and 2.24 g/L of sodium lactate. Both media were amended with 10 mM Mn(II) (~550 mg/L Mn) prepared from an MnSO_4_ stock solution. Ten milliliters of river water were inoculated into 100 mL of culture medium and incubated in an incubator set at 25 °C and 180 rpm. After 1 week of incubation, 10% (volume/volume) of each culture was transferred to fresh medium. Such sub-cultivations were performed 3 times. Each culture was then serially diluted and spread on the same type of agar medium amended with 10 mM Mn(II). These inoculated agar plates were incubated at 25 °C. After 5 days of incubation, colonies forming on these agar plates were selected and purified via continuous streaking on a fresh agar plate until a pure culture was obtained. The pure culture was transferred to a liquid medium and preserved for the subsequent experiment.

### 2.2. Manganese Removal by Five Isolated Bacteria

Five isolated bacteria were tested for their Mn removal performance. Bacteria were cultivated in the same medium from which they were isolated. The initial concentration of Mn(II) that was added to all cultures was 10 mM (~550 mg/L), and all bacterial cultures were incubated at 25 °C and 180 rpm. During the incubation, samples were extracted daily from all cultures to monitor changes in pH, optical density, and soluble Mn concentration. Biotic control for each isolate, wherein no Mn was added to the culture medium, and abiotic control, wherein no bacteria were inoculated, were run simultaneously for comparison.

### 2.3. Manganese Removal by Five Isolated Bacteria

The bacterium with the highest Mn removal efficiency (strain NDMn-6) was introduced to the subsequent experiment to investigate the effects of temperature and salinity. The bacterium was cultivated in the NB medium, following the experimental set-up summarized in Table 1. The cultivation temperature was investigated in the range of 20–40 °C, whereas the medium salinity was studied in the range of 0.1–5%. The salinity of medium was controlled by adding an appropriate amount of NaCl. In the experiment with various temperatures, the salinity of the culture medium was fixed at 0.5%, whereas in the experiment with various salinities, the cultivation temperature was maintained at 25 °C. In both experiments, the initial Mn concentration was set to 550 mg/L, and changes in pH, the optical density of the medium, and dissolved Mn concentration were monitored within 3 d of incubation.

### 2.4. Electron Microscopic Analysis and Characterization of Mn Precipitates

Strain NDMn-6 was cultivated in NB medium in the presence and absence of 10 mM Mn(II) to a stationary phase; cells were then harvested via centrifugation at 11,000 rpm for 15 min. Cells were fixed in glutaraldehyde 2.5% for 1 h, followed by osmium 1% for 1 h [20]. These specimens were then dehydrated by ethanol in incrementing concentrations ranging from 50–100%. The samples were finally treated with hexamethyldisilazane for enhancement of visibility under a microscope. These treated cells were dried on the surface of aluminum foil overnight in a desiccator under ambient temperature. Before being introduced to a field emission-scanning electron microscope (FE-SEM; SUPRA25, Zeiss, Oberkochen, Germany), the samples were coated with Pt for 100 s.

Strain NDMn-6 was cultivated with 10 mM Mn(II) in a large scale, at 4 L, to harvest precipitates for further characterization using X-ray diffractometer (XRD), FE-SEM, and energy-dispersive spectrometer (EDS) analyses. The Mn precipitate was harvested via centrifugation of the culture medium at 11,000 rpm for 15 min as described previously [17]. The sample was washed with distilled water and dried in a vacuum freeze-drying machine. The crystalline phase of precipitate powder was analyzed using the XRD system (X’pert3, Malvern Panalytical, Malvern, UK). The elemental composition of the precipitate was determined using an EDS equipment (EDAX Inc., Mahwah, NJ, USA) attached to the FE-SEM SUPRA25.

### 2.5. Analytical Methods

The changes in the pH of culture medium during the cultivation were quickly checked with a microvolume of the medium using a Compact pH meter LAQUAtwin-pH-22 (Horiba Scientific, Kyoto, Japan), as described previously [20]. The growth of bacteria was monitored through the optical density of the culture media at 600 nm (OD_600_) using a Spectro UV–Vis Double PC 8 Auto Cell Scanning Spectrophotometer (UV–Vis Double Beam Model UVD-3200, Labomed Inc., Los Angeles, CA, USA). The sample with OD_600_ value higher than 0.8 abs should be diluted, such that the recorded value was in the range of 0.3–0.8 abs. The medium supernatant harvested via centrifugation to remove cells and precipitate was filtered through a 0.2 µm syringe filter (Minisart, Gottingen, Germany) and diluted at an appropriate concentration for analyzing dissolved Mn concentration using inductively coupled plasma–atomic emission spectroscopy (ICP-AES; Activa, JY Horiba, Kyoto, France).

### 2.6. Phylogenetic Study of Isolated Strains

Pure colonies of isolated strains on agar plate were introduced to DNA extraction using HiGene^TM^ Genomic DNA Prep Kit (BIOFACT Co., Ltd., Daejeon, Korea) according to the manufacturer’s protocol. The genomic DNA was then used as a template for polymerase chain reaction (PCR) amplified 16S rRNA gene using universal primers 8 F and 1492R. The PCR experiment was conducted following a protocol that has been described previously [22]. The PCR products were then purified and sent to Solgent Co., Deajeon, Korea for sequencing. Single sequences were assembled and trimmed of low-quality initial and end regions using BioEdit v.7.5.0 (Ibis Biosciences, Carlsbad, CA, USA). The closest relatives of isolated strains were determined via a BLASTN search on the NCBI website. The phylogenetic tree of isolated strains and related sequences was constructed using MEGA X [23]. In this phylogenetic analysis, all sequences were aligned using ClustalW [24], the neighbor-joining method [25] was selected for phylogeny bootstrap test of 1000 replicates, and evolutionary distance was computed using the Jukes–Cantor method [26]. The pairwise deletion option was selected for missing or gap data in the nucleotide sequence collection. The 16S rRNA sequences of isolated strains NDMn-1, NDMn-2, NDMn-4, NDMn-6, and NDMn-7 were deposited in GenBank under accession numbers MT075557, MT075558, MT075559, MT075560, and MT075561, respectively.

## 3. Results and Discussion

### 3.1. Isolation of Mn Removal Bacteria

In the isolation process, a total of seven colonies (4 from BSM medium and 3 from NB medium) were selected and purified. However, after the sequencing, isolates NDMn-1 and NDMn-3 from the BSM medium were found to be the same, and isolates NDMn-5 and NDMn-6 from NB medium were found to be from the same strain. Therefore, five strains, including NDMn-1, NDMn-2, and NDMn-4 isolated from BSM medium, and NDMn-6 and NDMn-7 isolated from NB medium, were selected and maintained for the subsequent investigations. 

### 3.2. Cell Growth and Mn Removal by Five Isolated Bacterial Strains

**Figure 1:**
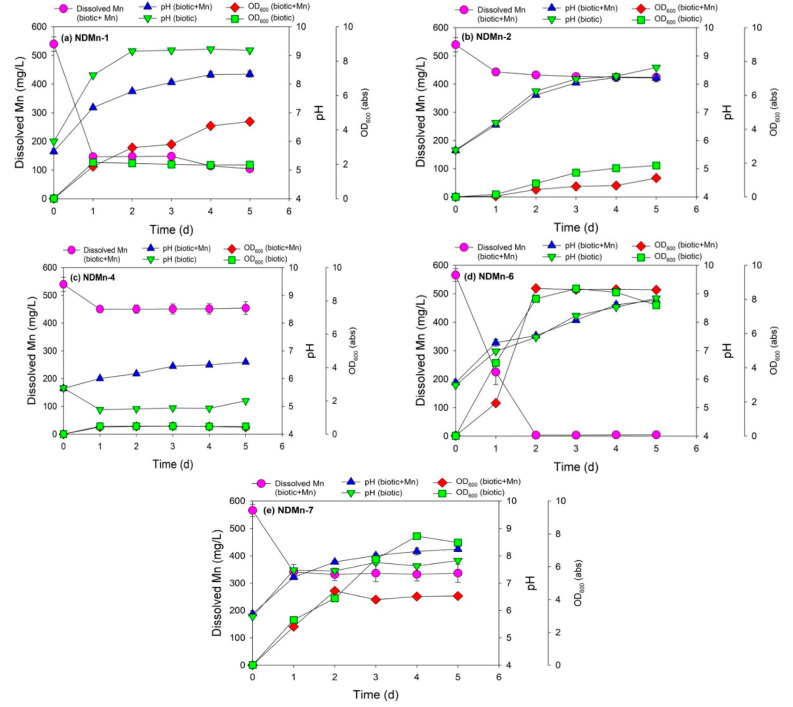
Manganese removal by strains (**a**) NDMn-1, (**b**) NDMn-2, (**c**) NDMn-4, (**d**) NDMn-6, and (**e**) NDMn-7, together with the biotic control of each strain.

Strain NDMn-1 was the bacterium that exhibited the highest Mn removal efficiency among the bacteria grown in BSM medium (strains NDMn-1, NDMn-2, and NDMn-4) (Figure 1a,b,c), whereas strain NDMn-6 was the better bacterium for Mn removal between two isolated strains from the NB medium (strains NDMn-6 and NDMn-7) (Figure 1d,e). Strain NDMn-1 could remove 73.1% soluble Mn from the culture medium after only 1 d of incubation (Figure 1a). Interestingly, the Mn removal efficiency did not significantly improve even with the incubation time extended up to 5 d. At the end of the incubation, Mn removal efficiency reached 80.6%. The optical density of the culture with Mn was much higher than that of the biotic control without Mn (4.5 abs versus 2.0 abs) (Table 2), which could be due to the presence of dispersive precipitated Mn particles in the culture medium. In the presence of Mn, the pH value of the culture medium was always lower than that in the absence of Mn during the incubation. The pH of both cultures increased significantly during the incubation (Figure 1a). The pH of the culture with Mn was stable at 8.3, whereas the pH of the biotic control culture without Mn reached a stable value of 9.2 after 2 d of incubation. This indicated that the basic compounds [27] secreted by strain NDMn-1 were consumed by the precipitation of Mn during the cultivation. The stability of dissolved Mn, pH, and OD_600_ in the abiotic control during the simultaneous incubation again validated that the removal of Mn in culture medium was a microbiological reaction (Supplementary Materials Figure S1).

The high pH value that resulted from bacterial growth could be a reason explaining the bioprecipitation of Mn in the culture of strain NDMn-1, according to Eh-pH diagram of Mn in water system [28]. However, this was not true with the observed changes in pH of culture strain NDMn-2 (Figure 1b), wherein the pH of both culture media with and without Mn reached around 8.6 at the end of incubation, whereas the Mn removal efficiency that was obtained was only 21.2%. Therefore, it can be said that the Mn removal is partially due to the pH increase but not entirely. Various fractions of Mn removal are involved in the metabolism of Mn(II)-oxidizing bacteria including oxidation, intracellular absorption, and extracellular adsorption [16,17]. The pH of NDMn-2 culture in the presence of Mn was close to that in the absence of Mn at all sampling points, whereas their OD_600_ values were slightly different. In contrast to that of strain NDMn-1, the OD_600_ of strain NDMn-2 without Mn was higher than that with Mn (1.9 abs vs. 1.1 abs), indicating that the cell density of strain NDMn-2 declined because of the addition of Mn to the culture medium. More interestingly, the presence of Mn resulted in a distinct difference in the pH changes of culture strain NDMn-4 during the incubation. The pH of culture strain NDMn-4 with Mn slightly increased, whereas that without Mn slightly decreased during the incubation, which created a significant difference in pH between both cultures (pH 6.6 versus pH 5.2) (Figure 1c). The OD_600_ of both cultures of NDMn-4 with and without Mn were stable at the same value (0.45 abs) during the incubation. The final Mn removal efficiency of strain NDMn-4 was 15.6%, which was the lowest among those of the three strains grown in BSM medium. Generally, strains NDMn-2 and NDMn-4 are not potential candidates for Mn removal; however, the presence of Mn in their culture resulted in something different in their culture properties, specifically the cell density of strain NDMn-2 and pH of strain NDMn-4.

Strain NDMn-6 grown in NB medium was found to be the best candidate for Mn removal. This bacterium could precipitate 99.1% soluble Mn in culture medium after only 2 d of incubation (Figure 1d and Supplementary Figure S2). The final dissolved Mn concentration in culture medium at the end of incubation was from 3–5 mg/L. Interestingly, the pH values of both culture strains of NDMn-6 with and without Mn were similar at all sampling points. The bacterial growth created an increase in the pH of the culture medium from 6.0 to 8.8 at the end of incubation. The OD_600_ results indicated that on the first day of incubation, the presence of Mn slightly affected bacterial growth, which resulted in a lower cell density; however, this influence was not maintained after 2 d of incubation. The cell density of culture without Mn was decremented after 3 d of incubation, whereas that of the culture with Mn was maintained stably at a high value (8.6 abs) until the end of incubation. Despite being grown in the same medium, strain NDMn-7 could remove only 40.2% Mn in the culture medium even with the incubation time extended up to 5 d (Figure 1e). The addition of Mn to the culture medium affected both the cell density and pH of strain NDMn-7, which were expressed through the significant difference in the changes of pH and OD_600_ from both cultures. The OD_600_ of the culture without Mn continuously increased until 4 d of incubation and was stable at 7.7 abs, whereas that of the culture with Mn reached a steady state after 2 d at 4.2 abs. In contrast to the OD_600_, the pH of the culture with Mn reached a higher value than that without Mn (8.2 vs. 7.6). Generally, the cell densities of a bacterial culture grown using NB medium were higher than those grown using BSM medium.

The Mn removal efficiency produced by strain NDMn-1 (80.6%) was lower than that produced by the recently reported strain DS02 (89.6%) [17], whereas strain NDMn-6 exhibited a much higher Mn removal efficiency (99.1%) than that of strain DS02 with the same initial Mn(II) concentration. A lower residual Mn in the final solution indicated that strain NDMn-6 was a better candidate for Mn removal and biogenic Mn oxides production. The Mn removal by both strains NDMn-1 and NDMn-6, especially, was much higher than by strain DS02 when cultivated with the same initial Mn(II) concentration (Table 3). Except for strain DS02, other previously reported strains presented high Mn removal efficiencies but low Mn removal rates because those bacteria could perform well at low Mn(II) concentrations (1 mM). *Lysinibacillus* spp. could produce Mn removal efficiencies of 65.5–94.7% and Mn removal rates of 4.0–7.4 mg/L/d [13,16]. *Brevibacillus* spp. produced high Mn removal efficiencies (83.6% and 94.0% for strains MO1 and MO2, respectively) when cultivated under an initial Mn(II) concentration of 1 mM. *Bacillus* spp. exhibited the lowest Mn removal efficiency and Mn removal rate among the recently reported species [13].

### 3.3. Cell Growth and Mn Removal by Strain NDMn-6 at Various Temperatures and Salinities

Strain NDMn-6 exhibited the highest Mn removal efficiency and Mn removal rate among the isolated bacteria and was determined to be a potential candidate for Mn removal and biogenic Mn oxides production. Therefore, its Mn removal performance was further investigated at various temperatures and salinities. These data would be useful for the future applications of this strain in field treatment.

**Figure 2:**
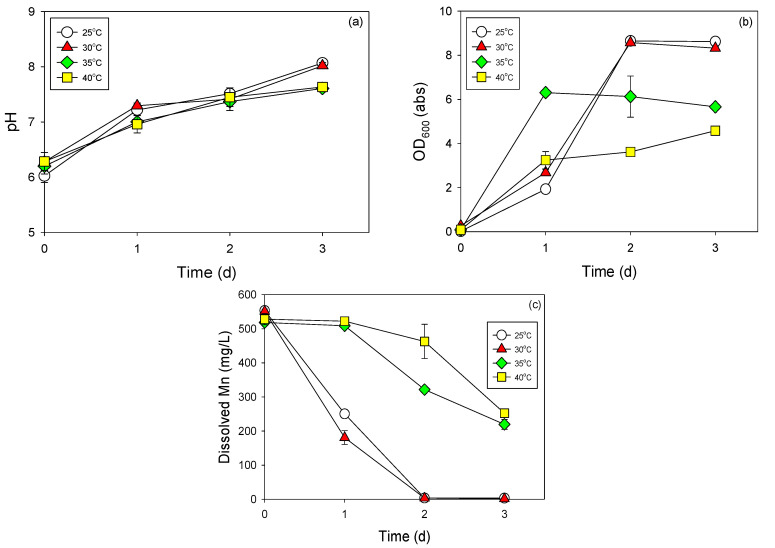
Manganese removal by strain NDMn-6 at various incubation temperatures. Changes in (**a**) pH (**a**), optical density (**b**), and dissolved Mn (**c**).

The obtained data revealed that strain NDMn-6 preferred an ambient temperature range (25–30 °C) for its growth and for Mn removal (Figure 2). No significant difference between the performance of strain NDMn-6 at 25 °C and 30 °C was found during observations of the cell growth (OD600), pH change, and the sink in soluble Mn concentration. The growth of strain NDMn-6 seemed to be slightly better at 30 °C compared to that at 25 °C after 1 d of incubation. However, the results after 2 d of incubation were the same at both temperatures. Acceleration of cell growth was observed at 35 °C through the data obtained at the sampling point at 1 d (Figure 2b). The OD_600_ of the culture incubated at 35 °C increased to 6.3 abs after 1 d, which is much higher than those at 25 °C and 30 °C. However, this acceleration in cell growth did not result in a good performance in terms of Mn removal (Figure 2c). The cell density of the culture at 35 °C was stable at steady state until the end of incubation. The inhibition of high temperature on the performance of strain NDMn-6 was observed and obvious at 40 °C. In this culture, the Mn removal could only be observed after 2 d of incubation. The pH variations during incubation at all temperatures were not significantly different (Figure 2a). The effect of environmental temperature on the performance of Mn-removing bacteria was not extensively investigated previously. The most recent studies reported the effect of temperature on the Mn-removal activities of *Brevibacillus brevis* MO1 and *Brevibacillus parabrevis* MO2 at 4 °C and 37 °C [19]. The results indicated that both bacteria performed better at 37 °C compared to at 4 °C.

**Figure 3:**
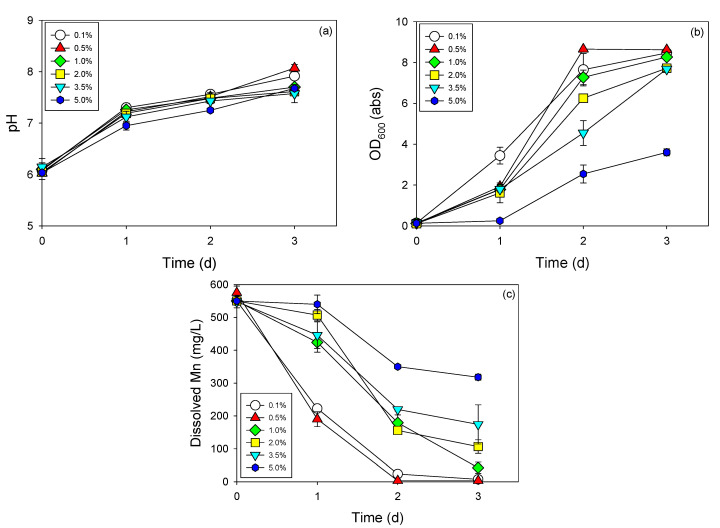
Manganese removal by strain NDMn-6 at various salinities. Changes in (**a**) pH, (**b**) optical density, and (**c**) dissolved Mn.

The effect of salinity on the activities of iron-oxidizing bacteria has been reported previously [29]. However, no up-to-date data are available regarding the influence of salinity on Mn removal by microbes. The results obtained in this study clearly indicated that Mn removal by strain NDMn-6 was substantially affected by the salinity of the culture medium (Figure 3). This was expressed through both the progression of OD_600_ (Figure 3b) and the decrement of soluble Mn (Figure 3c) in all cultures. No significant difference in the performance of strain NDMn-6 was observed in the salinity range of 0.1–0.5%. At a salinity of 1%, the Mn removal by strain NDMn-6 seemed to be delayed compared to those by the cultures at lower salinities. However, the Mn removal efficiency after 3 d was still acceptable. The strong influence of salinity on Mn removal efficiency was observed at a salinity range of 2–5%. However, this bacterial strain was recognized to be an extremely halotolerant bacterium because its growth could be observed after only 1 day of acclimation at salinities identical to that of seawater (3.5–5%). A considerably high Mn removal efficiency (80.7%) was observed at the end of incubation for the culture with 2% salinity. The Mn removal efficiencies after 3 d of incubation were 42.3% and 68.4% for cultures at salinities of 5% and 3.5%, respectively. The halotolerant characteristics of this strain were much better than those of some tellurite-reducing bacteria, which have been reported on recently [20]. The growths of both strains WAY and WYS were totally inhibited at a salinity of 5%. The results obtained in this study indicated that strain NDMn-6 could be applied for bioremediation of Mn in marine environments or for recovery of Mn from saline industrial wastewater.

### 3.4. Electron Microscopic Analysis and Characteristics of Mn Precipitates Produced by Strain NDMn-6

**Figure 4:**
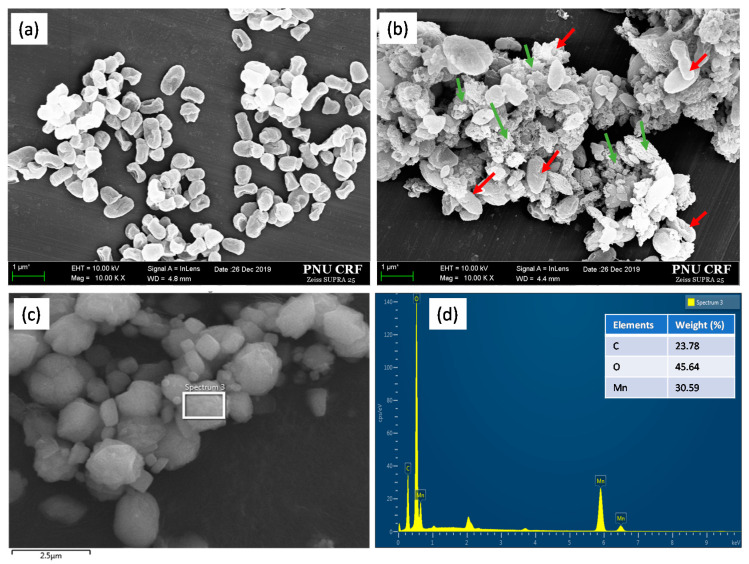
Electron microscopic analyses: SEM images of strains NDMn-6 growth (**a**) without Mn and (**b**) with Mn, at a magnification of 10,000×; (**c**) SEM image of Mn precipitates harvested from strain NDMn-6 culture, and (**d**) EDS spectrum of Mn precipitates. Red arrows indicate bacterial cells, and green arrows indicate Mn bioprecipitates.

**Figure 5:**
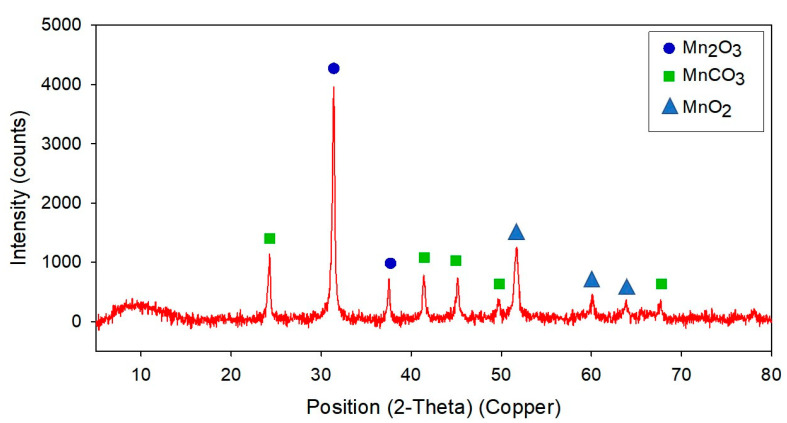
XRD pattern of Mn precipitates harvested from strain NDMn-6 culture.

Under a microscope, strain NDMn-6 cells are oval in shape, with lengths of 0.5–1.2 µm and diameters of 0.3–0.5 µm (Figure 4a). In the culture to which 10 mM Mn(II) was added, bacterial cells were found to occur together with Mn precipitated particles (Figure 4b). These biogenic Mn precipitates are in micro size, and their morphology is similar to those produced by *Acinetobacter* sp., which has been reported on previously [14]. The biogenic Mn powder harvested through the freeze-drying process was observed in various sizes, from 0.1–2 µm in diameter (Figure 4c). The elemental analysis by EDS revealed that those biogenic Mn precipitates contained 23.8% C, 45.6% O, and 30.6% Mn by weight (Figure 4d). The XRD patterns of the powder obtained in this study disclosed the presence of Mn_2_O_3_ crystal peak with a high intensity of up to 4000 counts (Figure 5). Furthermore, some significant peaks of MnCO_3_ (Rhodochrosite) were observed, and the peak of MnO_2_ was detected with low intensity. The XRD pattern of biogenic Mn powder obtained in this study was similar to that of biogenic Mn oxides from recent research [17]. This indicated that strain NDMn-6 is an Mn-oxidizing bacterium.

### 3.5. Phylogenetic Analysis of Isolated Strains

**Figure 6:**
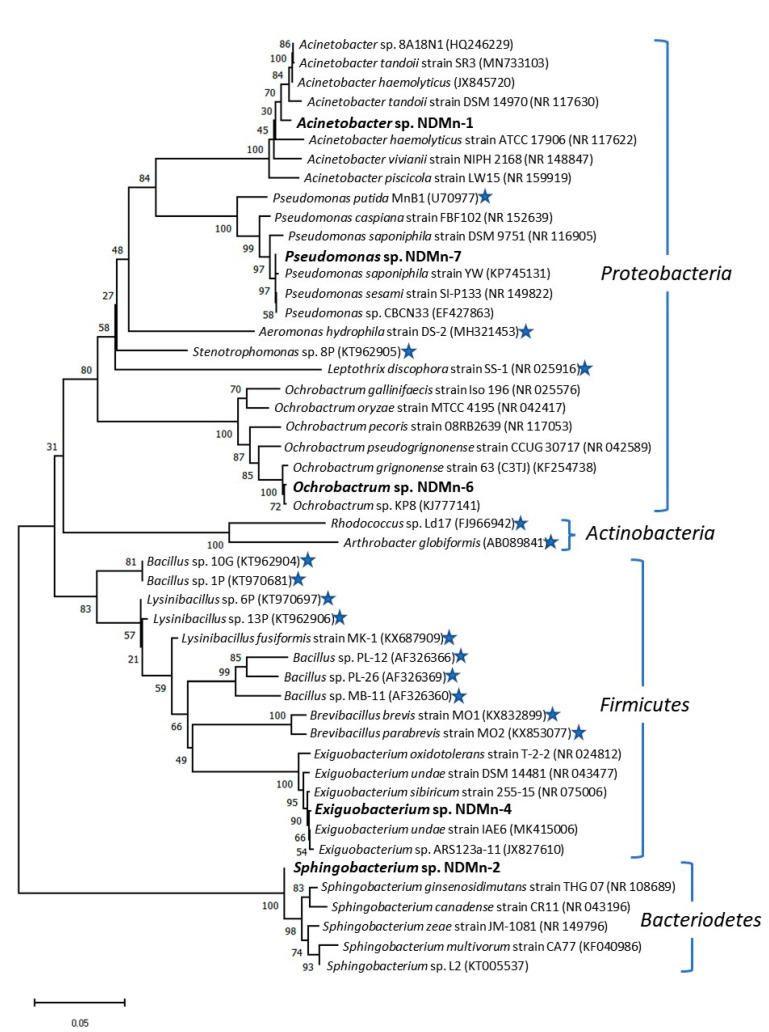
Phylogenetic tree showing the relationships of isolated bacteria with the closest relatives and previously reported Mn(II)-oxidizing bacterial strains. Bacterial strains in bold are isolated in this study. Bacterial strains with star mark are previously reported Mn(II)-oxidizing bacteria.

Five Mn-resistant bacteria isolated from Nakdong River water were determined to belong to three different bacterial phyla, including *Proteobacteria* (strains NDMn-1, NDMn-6, and NDMn-7), *Bacteroidetes* (strain NDMn-2), and *Firmicutes* (strain NDMn-4) (Figure 6). The 4 currently known strains affiliated with *Proteobacteria* include *Pseudomonas putida* MnB1 [30], *Aeromonas hydrophila* strain DS-2 [17], *Stenotrophomonas* sp. 8P [13], and *Leptothrix discophora* strain SS-1 [31]. Most of the other known Mn-oxidizing bacteria belong to *Firmicutes*, including *Bacillus* spp. [13,32], *Lysinibacillus* spp. [13,16], and *Brevibacillus* spp. [19]. *Rhodococcus* sp. Ld17 [33] and *Arthrobacter globiformis* [34] are two Mn-oxidizing bacterial strains belonging to the *Actinobacteria* phylum.

Strain NDMn-6 is affiliated with the *Ochrobactrum* cluster of *Alphaproteobacteria*, which is supported by a bootstrap value of 100%. The closest relative of strain NDMn-6 is *Ochrobactrum* sp. KP8, which shared a 99.8% sequence similarity with a 100% sequence coverage. Furthermore, strain NDMn-6 sequence also shared high sequence identities with *Ochrobactrum grignonense* strain 63 (C3TJ) (99.5%), *Ochrobactrum pseudogrignonense* strain CCUG 30,717 (97.7%), and *Ochrobactrum gallinifaecis* strain Iso 196 (96.2%). Based on the evolutionary analysis and sequence identity, strain NDMn-6 could be said to be representing a new strain of *Ochrobactrum* genus. As of now, this strain notably seems to be the first representative Mn-oxidizing bacterium from this genus.

Both strains NDMn-1 and NDMn-7 belong to the *Pseudomonadales* order of the *Gammaproteobacteria* class. However, strain NDMn-1 falls within the *Acinetobacter* genus cluster, whereas strain NDMn-7 is affiliated with the *Pseudomonas* genus cluster. Strain NDMn-1 shared 99.0% sequence similarity with *Acinetobacter* sp. 8A18N1 and *Acinetobacter haemolyticus* at 100% sequence coverage. Strain NDMn-1 could be concluded to have been a novel strain of the *Acinetobacter* genus. Although strain NDMn-7 shared 96.3% sequence similarity with a previously reported Mn-oxidizing bacterium, *Pseudomonas putida* MnB1 [30], the Mn removal performance of this strain was insignificant. Strain NDMn-7 shared 99.9% sequence identity with *Pseudomonas* sp. CBCN33, *Pseudomonas sesami* strain SI-P133, and *Pseudomonas saponiphila* strain YW at a sequence coverage of 100%, which confirmed that strain NDMn-7 was a strain of the *Pseudomonas* genus.

Strain NDMn-4 affiliated with the *Bacillales* order, which consists of many Mn-oxidizing bacteria, including *Bacillus* spp. [13,32], *Lysinibacillus* spp. [13,16], and *Brevibacillus* spp. [19]. However, the Mn removal performance of this strain was negligible. This strain shared 99.8% sequence similarity with *Exiguobacterium undae* strain IAE6 (MK415006), which is its closest relative. Strain NDMn-4 also shared high sequence identities with *Exiguobacterium* sp. ARS123a-11 (99.7%), *Exiguobacterium sibiricum* strain 255-15 (99.7%), and *Exiguobacterium undae* strain DSM 14,481 (99.2%). Strain NDMn-2 represented a new branch within the *Sphingobacterium* genus cluster. This strain shared high sequence similarities with different species of this genus, including *Sphingobacterium multivorum* strain CA77 (99.6%), *Sphingobacterium ginsenosidimutans* strain THG 07 (99.3%), *Sphingobacterium* sp. L2 (99.2%), *Sphingobacterium canadense* strain CR11 (99.2%), and *Sphingobacterium zeae* strain JM-1081 (99.1%). Strain NDMn-2 was the first Mn-resistant strain of the *Sphingobacterium* genus.

## 4. Conclusions

This study provided a novel bacterial strain with a high Mn removal rate for application in Mn removal from contaminated water sources. Strain NDMn-6 exhibited the highest Mn(II) removal efficiency (99.1%) among five bacteria isolated from Nakdong Tiver. This strain was determined to be *Ochrobactrum* sp. NDMn-6, which is an Mn-oxidizing bacterium of the *Alphaproteobacteria* class. The final product produced through Mn precipitated from aqueous phase was defined as a mixture of Mn_2_O_3_, MnO_2_, and MnCO_3_, which was considered a potential material in environmental remediation and industries. The strain could optimally perform Mn removal at temperatures of 25–30 °C and salinities of 0.1–0.5%. This strain can especially tolerate a salinity of up to 5% and even produce a high Mn removal efficiency at a salinity of 2%. This halotolerant bacterium could be considered as a candidate for the Mn removal from saline wastewater or Mn-contaminated brackish water regions.

## Figures and Tables

**Table 1:** Experimental set-ups for optimization of Mn(II)-oxidation by strain NDMn-6.

Environmental Factors	Temperature	Salinity
Various temperature experiment	20 °C	0.5%
	25 °C	
	30 °C	
	37 °C	
	40 °C	
Various salinity experiment	25 °C	0.1%
		0.5%
		1.0%
		3.5%
		5.0%

**Table 2:** Mn removal by five isolated bacteria.

Bacterial Strains	Culture Media	Final Mn Concentration (mg/L)	Final pH (with Mn)	Final pH (without Mn)	Final OD_600_ (with Mn, abs)	Final OD_600_ (without Mn, abs)
NDMn-1	Basal salt	104.48 ± 5.13	8.35 ± 0.11	9.18 ± 0.02	4.49 ± 0.07	1.97 ± 0.01
NDMn-2	Basal salt	424.43 ± 6.68	8.22 ± 0.14	8.59 ± 0.05	1.12 ± 0.01	1.86 ± 0.03
NDMn-4	Basal salt	454.35 ± 18.80	6.60 ± 0.06	5.20 ± 0.11	0.41 ± 0.00	0.47 ± 0.01
NDMn-6	Nutrient broth	4.52 ± 0.78	8.77 ± 0.06	8.84 ± 0.09	8.56 ± 0.02	7.67 ± 0.02
NDMn-7	Nutrient broth	336.71 ± 27.12	8.25 ± 0.09	7.83 ± 0.05	4.22 ± 0.01	7.48 ± 0.01

**Table 3:** Comparison of Mn removal efficiencies and Mn removal rates by some recently reported strains.

Bacterial Strains	Mn Removal Efficiency (%)	Mn Removal Rate (mg/L/d)	Reference
*Lysinibacillus* sp. MK-1	94.7	7.4	[16]
*Brevibacillus brevis* MO1	83.6	4.6	[19]
*Brevibacillus parabrevis* MO2	94.0	5.2	
*Acinetobacter* sp.	90.0	7.1	[14]
*Aeromonas hydrophila* strain DS02	89.6	82.1	[17]
*Bacillus* sp. 1G	58.5	4.0	[13]
*Bacillus* sp. 10G	63.0	4.4	
*Stenotrophomonas* sp. 7P	70.9	5.0	
*Stenotrophomonas* sp. 8P	66.4	4.6	
*Lysinibacillus* sp. 6P	65.6	4.0	
*Lysinibacillus* sp. 6P	82.7	5.3	
*Acinetobacter* sp. NDMn-1	80.6	147.7	This study
*Ochrobactrum* sp. NDMn-6	99.1	181.7	

## Supplementary Materials

The following are available online at https://www.mdpi.com/2218-273X/10/6/941/s1, Figure S1: Changes of dissolved Mn, pH, and OD of abiotic control basal salt medium (a) and nutrient broth medium (b), Figure S2: Comparison of Mn removal efficiency by five isolated strains.
