# Supplementary material for: Biological Manganese Removal by Novel Halotolerant Bacteria Isolated from River Water
Source: Biomolecules. 2020 Jun 22;10(6):941. doi: 10.3390/biom10060941 (PMC7356865; doi:10.3390/biom10060941)
Supplement: Supplementary file 1 [file biomolecules-10-00941-s001.pdf]

Supplementary Materials

Biological manganese removal by novel halotolerant bacteria isolated from river water

Van Khanh Nguyen [1,2], Myung-Gyu Ha [3], Ho Young Kang [4] and Dinh Duc Nguyen [5,*]

[1] Laboratory of Advanced Materials Chemistry, Advanced Institute of Materials Science, Ton Duc Thang University, Ho Chi Minh City, Vietnam; nguyenvankhanh@tdtu.edu.vn
[2] Faculty of Applied Sciences, Ton Duc Thang University, Ho Chi Minh City, Vietnam
[3] Korea Basic Science Institute, Busan Center, Busan 46742, Korea; mkha@kbsi.re.kr
[4] Department of Microbiology, Pusan National University, Busan 46241, Korea; hoykang@pusan.ac.kr
[5] Institution of Research and Development, Duy Tan University, Da Nang 550000, Vietnam
* Correspondence: nguyendinhduc2@duytan.edu.vn (D.D.N.)

Pages: 3

Figures: 2

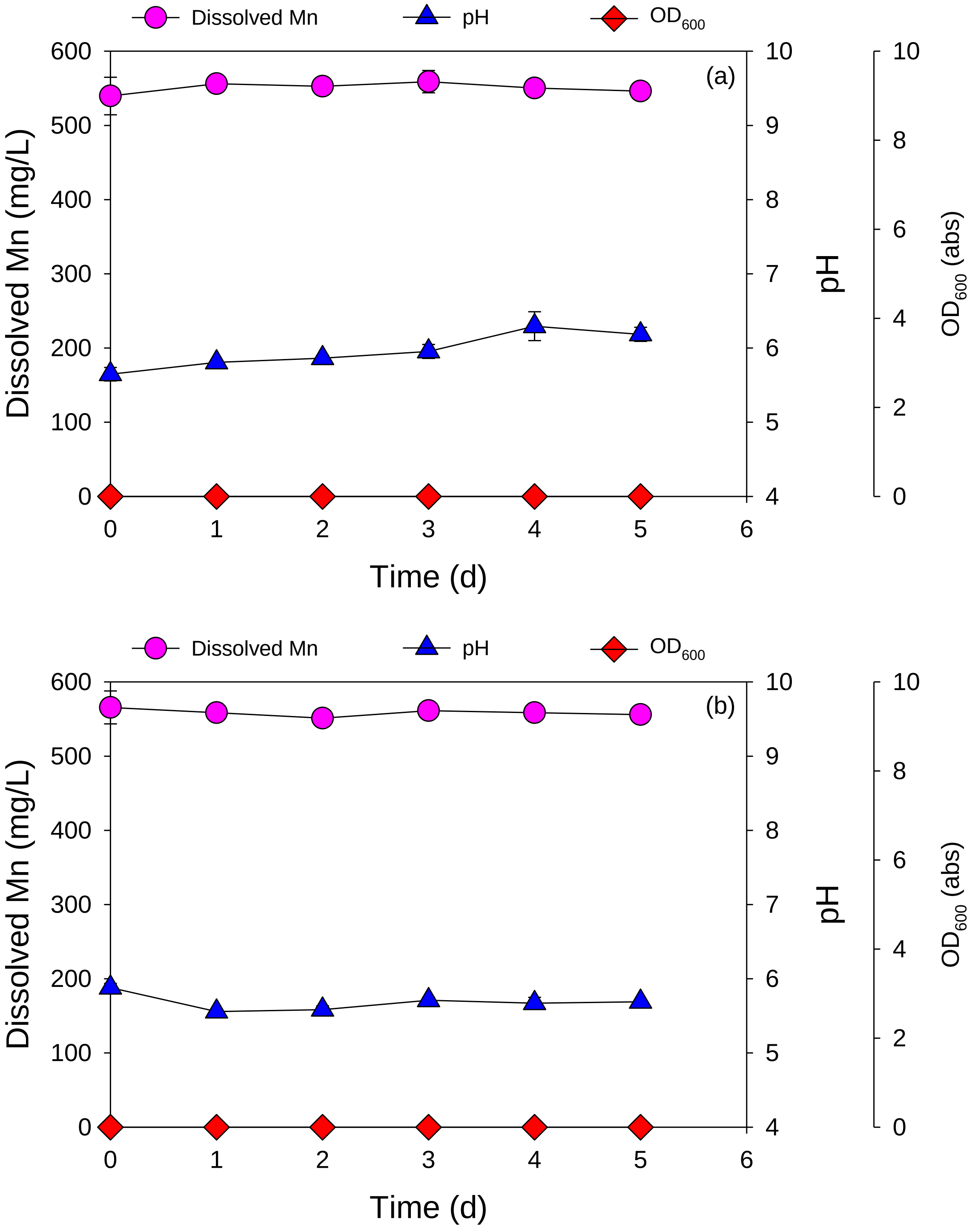

Fig. S1 Changes of dissolved Mn, pH, and OD of abiotic control basal salt medium (a) and nutrient broth medium (b).

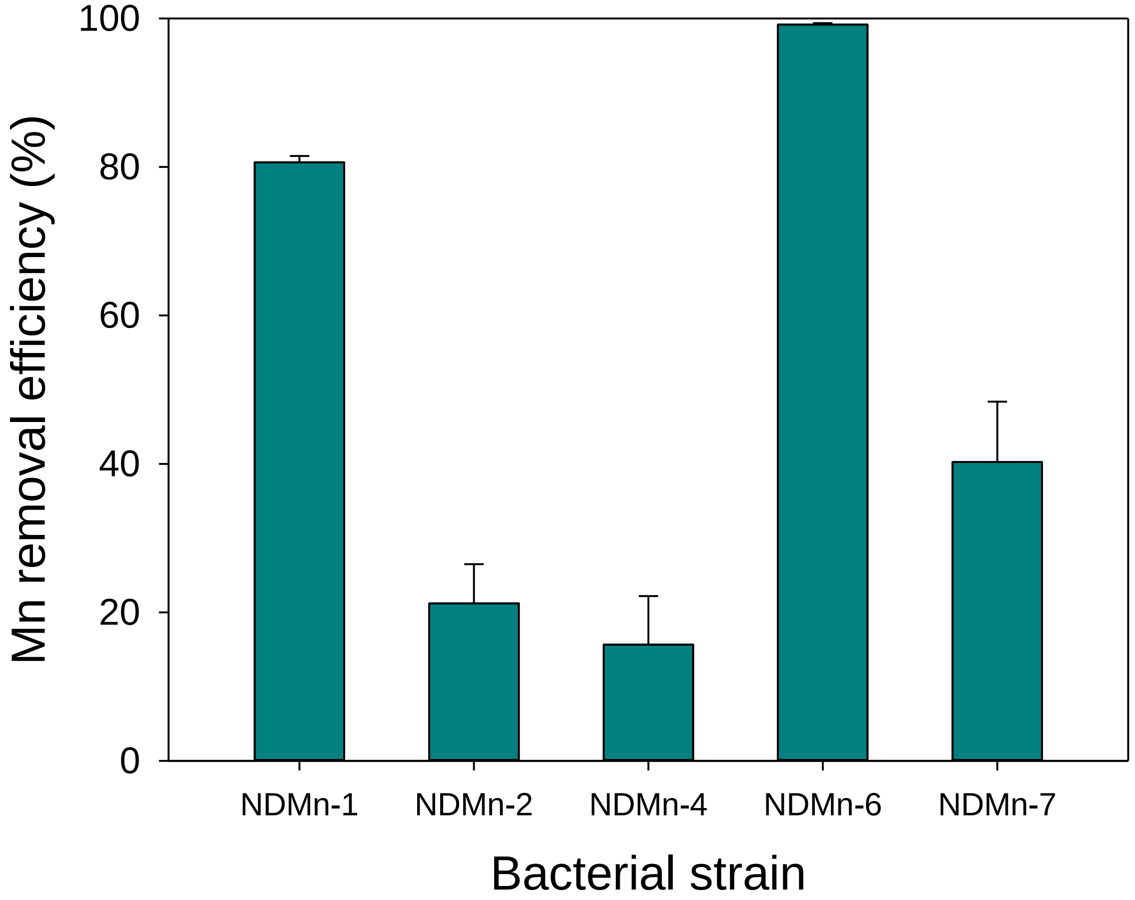

Fig. S2 Comparison of Mn removal efficiency by five isolated strains
